# Fractal Pattern in the Premature Graying of Hair: A Case Report

**DOI:** 10.7759/cureus.59994

**Published:** 2024-05-09

**Authors:** Brandy Phan, Amna M Ali, Troy A Black, Alisha Kashyap, Maryam Niazi, Rashid M Rashid

**Affiliations:** 1 Dermatology, University of Texas Health Science Center at Houston McGovern Medical School, Houston, USA; 2 Dermatology, Texas Tech University Health Sciences Center School of Medicine, Lubbock, USA; 3 Dermatology, Mosaic Dermatology, Houston, USA

**Keywords:** white hair, gray hair, premature graying of hair, premature aging, canities

## Abstract

Premature graying of hair (PGH) is a multifactorial condition defined by the graying of hair before the age of 20 in Caucasians and before the age of 30 in African Americans. Although the etiology remains unknown, it has been associated with genetic predisposition, oxidative stress, nutritional deficiencies, and autoimmune diseases. Current treatment options are limited but can include anti-inflammatory medications, vitamins, and hair colorants for symptom control. In this report, we present a case of premature graying in a 32-year-old male, onset at age 15, exhibiting a distinctive fractal pattern. This case represents a unique instance of PGH characterized by an unusual pattern, necessitating further investigation into potential etiological factors and underlying pathophysiological mechanisms.

## Introduction

Premature graying of hair (PGH), also known as premature canities, is characterized by the early onset of gray or white hair. The age indicating PGH is primarily determined by ethnicity, with Caucasians meeting the criteria if graying appears before the age of 20 and Africans before the age of 30. While the graying of hair is a natural part of aging, the existing literature indicates premature graying as a multifactorial condition influenced by factors, including genetic predisposition, oxidative stress, nutritional deficiencies, and tobacco exposure [1]. Although research has made strides in understanding the microscopic, biochemical, and molecular alterations occurring within the hair follicle, the precise mechanism behind premature graying remains elusive, thereby hindering treatment options [2]. This report outlines a case of premature graying in a 32-year-old male, which commenced at the age of 15 and exhibits a unique fractal pattern.

## Case presentation

**Figure 1:**
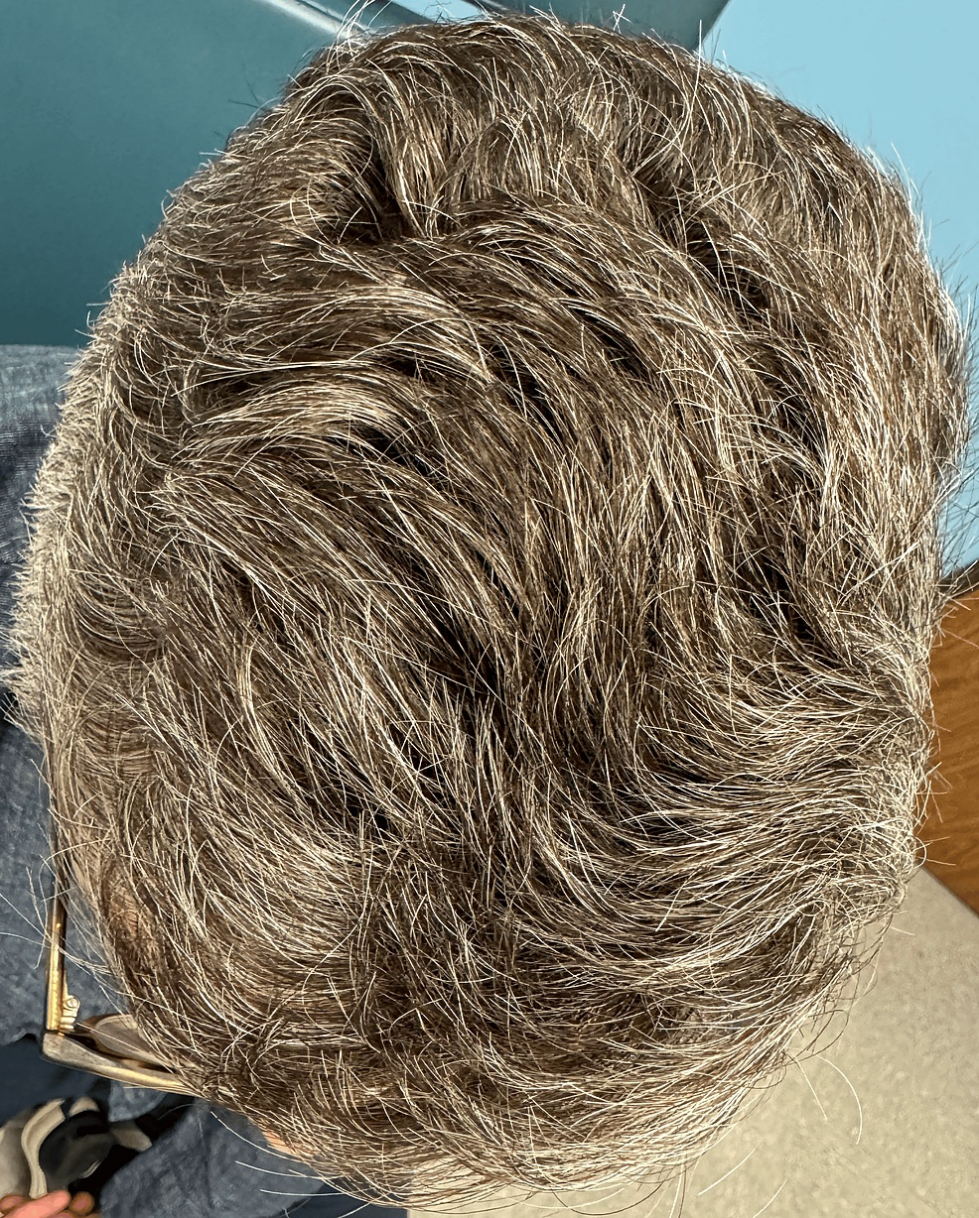
Distinctive pattern of gray hair distribution observed on the scalp

**Figure 2:**
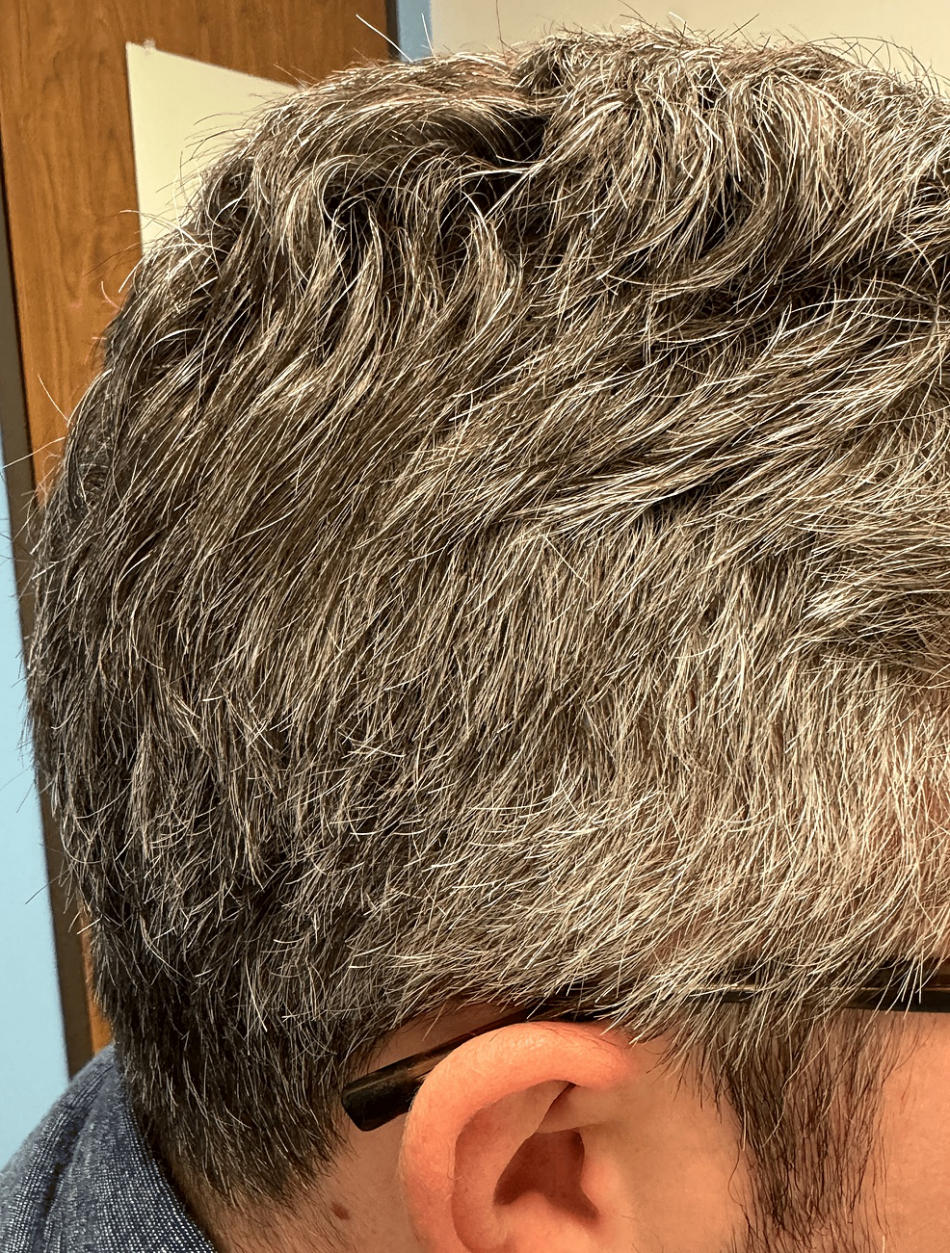
Progression of gray hair distribution to the right temporal area

A 32-year-old Turkish Caucasian male presented to the dermatologist for the evaluation of premature graying. He began experiencing the onset of gray hair on his face and scalp at the age of 15. The progression has been gradual and remained stable over recent years. Physical exam revealed a distinctive pattern of graying hair resembling a wave-like or fractal arrangement (Figures 1, 2). The patient reported no significant past medical or family history. Furthermore, the patient's bloodwork was normal with no nutritional deficiencies noted. Due to the unique pattern present in this case, further investigation into potential etiological factors and underlying pathophysiological mechanisms was warranted.

## Discussion

Due to variations in the structural characteristics across different ethnicities, the aging process of hair can vary extensively (Table 1). A study by Panhard et al. revealed that African, Thai, and Chinese individuals with darker hair tones tend to experience gray hair less frequently and with less intensity compared to individuals with lighter hair shades, such as Caucasians, who are of similar ages [3]. More specifically, the incidence of graying among sub-Saharan African individuals was found to be 43%; among Thai individuals, 67%; and among Chinese individuals, 69%. Comparatively, the incidence of graying among European-derived Caucasian individuals was found to average 82% [3]. The average age of onset for hair graying varies by ethnicity, with Caucasians generally starting to gray in their mid-30s, Asians in their late 30s, and Africans in their mid-40s [4]. The later onset of graying in African populations is thought to be attributed to their larger melanosomes and increased melanocyte density when compared to individuals of Caucasian and Asian descent [4]. Ji et al. reported that hair among Asian ethnic groups has higher levels of integral lipids, fatty acids, cholesterols, and waxy esters as compared to Caucasian and African populations, decreasing its susceptibility to oxidative stress from ultraviolet (UV) damage [5]. Age-related changes noted in the hair follicles of all ethnicities include a decrease in the production of eumelanin and an increase in susceptibility to oxidative stress [4].

**Table 1:** Summary of hair characteristics by ethnicity

	Below average	Above average
Incidence of graying		
African, Thai, and Chinese individuals	x	
Caucasian individuals		x
Age of onset of graying		
Caucasian individuals	x	
Asians and African individuals		x
Hair susceptibility to oxidative stress		
Asian individuals	x	
Caucasian and African individuals		x

The impact of reactive oxygen species (ROS) on hair graying has been extensively explored in the literature, with most concluding that the accumulation of ROS leads to graying of hair. In the active growth phase (anagen), melanogenesis within the hair follicle is vigorous. This process entails the hydroxylation of tyrosine and the oxidation of dihydroxyphenylalanine to produce melanin, which results in significant cumulative oxidative stress. Causes of oxidative stress include UV rays, pollution, emotional factors, and inflammatory causes. Cigarette smoking also generates significant levels of reactive oxidative species, damaging melanocytes [6]. Insufficient expression of antioxidants, such as catalase and methionine sulfoxide reductase, may impair melanocyte function, consequently leading to diminished pigmentation and the emergence of gray hair follicles [1].

Nutritional deficiencies, including lower levels of vitamin B12, copper, iron, calcium, and vitamin D3, are also thought to play a role in PGH through the modulation of various metabolic processes [7]. For example, it is proposed that iron modulates tyrosinase activity as the enzyme dopachrome tautomerase, which is responsible for a later stage of melanin biosynthesis, is a metalloenzyme that utilizes ferrous, a form of iron, in its active site [8]. Sharma and Dogra found that patients with PGH reported a significantly higher incidence of atopic diathesis and a sedentary lifestyle, as well as significantly lower levels of high-density lipoprotein (HDL)-cholesterol [9]. Additionally, autoimmune disorders such as vitiligo, pernicious anemia, autoimmune thyroid disease, and premature aging syndromes, such as Werner's syndrome, are also found to be associated with PGH [7].

The incidence of PGH is found to be comparable between males and females; however, there appear to be significant differences in the clinical presentation of PGH. Jo et al. reported that the temporal and occipital areas are more commonly involved in males when compared to females. Moreover, graying typically begins in the temporal area in males but in the frontal area in females. Age of onset is also associated with variability in presentation; patients with earlier-onset PGH report more involvement of the parietal and occipital areas, while those with later-onset PGH report more frontal area involvement [10].

Currently, treatment options for PGH are limited. Physicians have explored the experimental use of various medications for PGH, with numerous case reports or series detailing their findings as they sought potential efficacy in their patients. Some reported medications include anti-inflammatory medications, stimulators of melanogenesis, and vitamins such as calcium pantothenate or potassium para-aminobenzoate (PABA). Treatment can also be tailored to address the presumed underlying cause of PGH, especially in patients with hormonal or nutrient deficiencies. A majority of patients continue to rely on camouflage techniques, such as hair colorants, for desired symptom management [11].

## Conclusions

While the etiology of premature graying remains poorly understood, ethnic variations in hair structure and susceptibility to oxidative stress seemingly play significant roles. Clinically, this case reemphasizes the importance of a personalized approach to diagnosis and management, considering individual patient characteristics and potential underlying factors. Further research is needed to understand the precise mechanisms of premature graying and develop targeted therapeutic interventions. Understanding these clinical implications can help guide healthcare providers in delivering comprehensive care to those affected by premature graying, ultimately improving outcomes and quality of life.
